# Special Issue “The Prevention and Treatment of Atherosclerosis”

**DOI:** 10.3390/jcm11041023

**Published:** 2022-02-16

**Authors:** Anna Kabłak-Ziembicka

**Affiliations:** 1 Departament of Interventional Cardiology, Institute of Cardiology, Jagiellonian University Medical College, 31-008 Kraków, Poland; kablakziembicka@op.pl; 2 John Paul II Hospital, Prądnicka 80, 31-202 Kraków, Poland

This editorial summarizes the 10 scientific papers that contributed to the Special Issue of the *Journal of Clinical Medicine*: ‘The Prevention and Treatment of Atherosclerosis’.

Papers published in this Special Issue focused on the biomarkers of cardiovascular diseases and atherosclerosis-associated risk factors and comorbidities.

Arterial properties such as vascular stiffness, carotid intima-media complex thickening (CIMT), and plaque formation are important, as non-traditional cardiovascular risk factors associated with risk of fatal and non-fatal cardiovascular events such as myocardial infarction, stroke, and heart failure episodes. Four papers addressed this clinically relevant problem [1,2,3,4].

In a cohort group of 404 patients with cardiovascular risk factors, including 267 patients with moderate-to-severe degenerative aortic valve stenosis, increased values of vascular stiffness (i.e., resistive and pulsatile indices) were associated with 21% to 25% risk increase of heart-failure episodes and major adverse cardiac and cerebral events at 2.5 years follow-up [1].

Formanowicz et al. proposed, based on the self-constructed logistic model of the growth of CIMT, that the optimal patient age for starting the statin treatment is when the CIMT growth curve is at its steepest part on the S-shape curve. Such an approach would allow to prevent CIMT from further thickening, hopefully preventing adverse cardiovascular events [2]. This is in line with the conception of the non-linear CIMT growth, with periods of attenuated and rapid progression described in the review paper of this Special Issue [3]. CIMT, once the measurement approach is unified, is a promising tool to assess the cardiovascular risk in both primary and secondary care patients [3]. Furthermore, markers of subclinical atherosclerosis, including CIMT, pulse wave velocity, aortic and brachial augmentation indexes, and aortic systolic blood pressure, all are associated with four main CV risk scores: SCORE, Framingham, QRISK, and PROCAM in the asymptomatic population [4].

Statin therapy was also addressed in the study by Ichikawa et al., who evaluated the association of serum malondialdehyde low-density lipoprotein (MDA-LDL), an oxidatively modified LDL, with the prevalence of high-risk plaques determined with coronary computed tomography angiography (CTA) in statin-treated patients [5]. The authors of this publication found that a high serum MDA-LDL level is an independent predictor of CTA-verified high-risk plaques, which, despite statin treatment, can lead to cardiovascular events [5].

Similarly, in a very elegant review paper, features of high-risk plaques were addressed in patients with myocardial infarction but non-obstructive coronary artery disease [6]. This condition, despite a lack of stenotic and occlusive lesions in the culprit coronary artery, is associated with a high mortality rate, varying between 2.2% and 3.9% at 12 months follow-up [6]. Due to the high resolution of optical coherence tomography (OCT), OCT displays plaques with high-risk features, such as erosion, thin fibrous cap, ruptured, or large lipid core, as well as enabling to differ between plaque disruption, spontaneous coronary artery dissection, coronary artery spasm, and coronary thromboembolism, which can drive optimal management focused on reducing the morbidity and mortality in that subset of patients [6].

Atherosclerosis is not solely a lipid condition but is also a consequence of local blood coagulation activation that takes place inside atherosclerotic lesions and contributes to their growth. The imbalance between thrombin-mediated fibrin formation and fibrin degradation might enhance atherosclerosis in relation to inflammatory states reflected by increased fibrinogen concentrations, the key determinant of fibrin characteristics [7]. As evidenced, the formation of a dense fibrin structure, which is resistant to lysis, increases patients’ risk of atherosclerosis progression and thromboembolic events. Many traditional cardiovascular risk factors, including hyperlipidemia, hypertension, smoking, and diabetes, are associated with altered fibrin clot properties [7]. Active treatment of cardiovascular risk factors increases the fibrin clot permeability and facilitates its lysis [7].

Authors of the next paper searched for the mechanisms of smoking toxicity in patients with peripheral arterial disease (PAD) [8]. Their data suggest that a downregulation of miR-27b mediates the proatherogenic effects of cigarette smoking on the incidence and severity of PAD, which may be attenuated by smoking cessation [8].

Sabatel-Perez et al. presented their strategy for improving familial hypercholesterolemia (FH) detection through the centralized analytical database, which can increase the diagnostic percentage of FH from 5.3% before to 12.2% after the screening strategy was applied [9]. The early detection of FH is important to start and optimize treatment, which greatly reduces the risk of atherosclerotic cardiovascular disease, and perform family-based cascade screening [9].

Finally, the very fashionable topic of vitamin D deficiency, which in the worldwide population may have multiple effects on the cardiovascular system, was explored [10]. The authors found low levels of vitamin D in patients without significant changes within the coronary arteries, and even lower vitamin D levels in patients with single-, double-, or triple-vessel coronary artery disease [10]. Severe (<10 ng/mL) and moderate vitamin D deficiency (≥10 to <20 ng/mL) were observed in 74% of patients with significant coronary artery disease [10].

Overall, these 10 contributions published in this Special Issue further strengthen the relationship between subclinical atherosclerosis, imaging and serum biomarkers, mechanisms of atherosclerosis progression and evolution, and incidence of adverse cardiovascular events.
